# Determinants of Leukocyte Margination in Rectangular Microchannels

**DOI:** 10.1371/journal.pone.0007104

**Published:** 2009-09-21

**Authors:** Abhishek Jain, Lance L. Munn

**Affiliations:** 1 Steele Lab for Tumor Biology, Massachusetts General Hospital, Harvard Medical School, Charlestown, Massachusetts, United States of America; 2 Department of Biomedical Engineering, Boston University, Boston, Massachusetts, United States of America; Sun Yat-Sen University, China

## Abstract

Microfabrication of polydimethylsiloxane (PDMS) devices has provided a new set of tools for studying fluid dynamics of blood at the scale of real microvessels. However, we are only starting to understand the power and limitations of this technology. To determine the applicability of PDMS microchannels for blood flow analysis, we studied white blood cell (WBC) margination in channels of various geometries and blood compositions. We found that WBCs prefer to marginate downstream of sudden expansions, and that red blood cell (RBC) aggregation facilitates the process. In contrast to tubes, WBC margination was restricted to the sidewalls in our low aspect ratio, pseudo-2D rectangular channels and consequently, margination efficiencies of more than 95% were achieved in a variety of channel geometries. In these pseudo-2D channels blood rheology and cell integrity were preserved over a range of flow rates, with the upper range limited by the shear in the vertical direction. We conclude that, with certain limitations, rectangular PDMS microfluidic channels are useful tools for quantitative studies of blood rheology.

## Introduction

Leukocyte margination from the center of a blood vessel toward the vascular endothelium is an important process in the inflammatory response and is affected by many factors. In 1961, Williamson *et. al.* showed by electron microscopy that WBCs accumulate at the vessel wall during acute inflammation [1]. Cell-cell interactions within the flow stream determine the frequency of leukocyte-endothelium collisions and after contact, adhesion molecules control leukocyte rolling, firm adhesion and emigration [2]–[4]. *In vitro* experiments assessing the radial distribution of white blood cells in small glass tubes (69 µm to 200 µm diameter) have shown that margination may depend on rheological factors such as hematocrit, blood suspension medium and shear stress [5]–[9]. Studies *in vivo* have shown that erythrocyte aggregation has a major influence on margination of white blood cells and platelets [10]–[12]. WBC margination in large rectangular channels (3 mm wide and 300 µm deep) also shows dependence on blood rheology [13]. In addition to intrinsic rheological properties, the size and geometry of the conduit plays an important role in margination [5], [14]–[16]. It has been established that leukocytes preferentially roll and adhere to the endothelium in postcapillary venules [4], [5], [10]. The increased leukocyte activity in these regions is maintained by adhesion molecules such as selectins [17]–[19], VCAM-1, and ICAM-1 [17], [20], [21], but the initiation of rolling requires margination and wall contact, which is a result of the specific fluid dynamics in the expanding conduit [5], [10]. Computational studies have been used to dissect the cell-cell interactions and the role of RBC aggregation in expanding channels [4], [22].

Initial pioneering studies of blood cell dynamics *in vitro* were performed in glass tubes or other simple geometries [23]–[28]. A few early studies used large-scale systems, which were more convenient and controllable [5], [7], [9]. Many advances have also been made using parallel plate flow chambers to study the dynamics of cell-surface adhesion [29], [30]. But it has been difficult to adapt these simple systems to the study of blood flow dynamics in more complex, representative geometries.

Advances in microfabrication technology have provided useful tools for studying blood flow and cell interactions in artificial networks with complex topology, at the scale of the microvasculature [31]–[33]. Recent studies have shown these microchannels can be used to study the microcirculation [33] as well as create practical devices for blood separation [34]–[37]. Networks of microfluidic channels can be constructed based on real network structures with practically any level of complexity desired. But these devices also have potentially important limitations. Perhaps the most important is that current PDMS molding technology produces systems of channels that are rectangular in cross-section; structures with circular cross sections are difficult to fabricate using standard photolithography and molding. Thus, before they can be used as surrogates for real micro-vessel networks, the implications of the rectangular geometries must be understood. To date, there are no quantitative studies comparing blood dynamics in rectangular microchannels and circular microvessels.

In this study, our aim was to characterize WBC margination in microchannels of various geometries. Specifically, we quantified how flow conditions, fluid composition, conduit size and channel geometry affect leukocyte margination in microchannels molded in PDMS. Our results are discussed in the context of previous experiments using real vessels or circular tubes.

## Results

To assess the dynamics of blood in pseudo 2D rectangular microchannels, we performed experiments in which we varied 1) the suspending media composition 2) channel size and geometry (width (*w*) and depth (*d*)), 3) shear rate along the channel width (*γ_w_*) and 4) hematocrit (*H_o_*).

**Figure 1:**
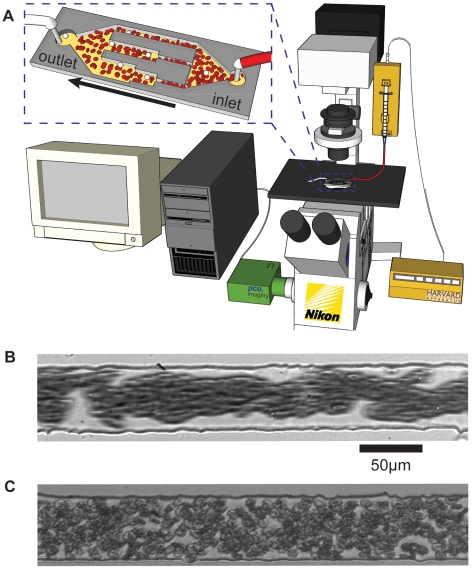
Experimental setup and erythrocyte aggregation in rectangular microchannels. A) illustration of experimental setup. Blood is perfused through the PDMS devices via a syringe pump. Labeled WBCs are visualized using a high speed CMOS camera attached to an epifluorescence microscope. Images are recorded on a PC using Camware V2.22 software and analyzed using Matlab. B) Flow of RBCs (*H_o_* = 10%) in a 50 µm wide channel suspended in 2% Dextran 500 solution; note the prominent plasma rich layer C) In RPMI 1640 medium, RBCs touch the wall and plasma rich zone is minimal. Flow rate and hematocrit are the same for (B) and (C).

It is well known that RBC aggregation plays an important role in margination in tubes, so we used various blood compositions that either enhanced or inhibited RBC aggregation. RBCs form aggregates when suspended in large polymer solutions such as Dextran which effectively cross-link the RBCs [38]–[40].Therefore, we used high molecular weight (MW) Dextran (MW:500 kD) to enhance RBC aggregation (Figure 1B), and plasma-free culture medium (RPMI-1640) to minimize it (Figure 1C). Autologous plasma (platelet-depleted) was also used to re-constitute the blood, since it also supports aggregation and is more physiologically-relevant. To examine how conduit geometry affects blood cell dynamics, we compared WBC margination in channels with constant or varying width. The effects of aggregation, *H_o_*, *γ_w_*, and channel geometry on leukocyte margination were quantified by recording the percentage of leukocytes contacting the sides of the channel at given locations along the channels (%WBC).

### WBC margination in straight channels

**Figure 2:**
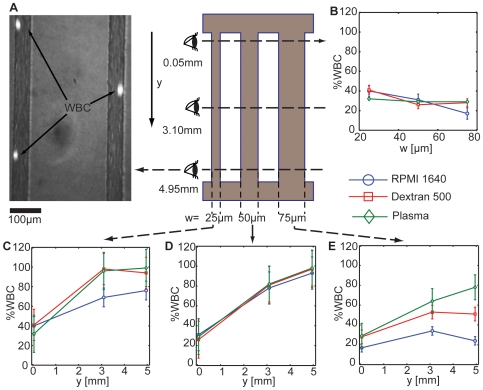
The number of WBCs contacting the wall depends on the distance from the inlet (*y*), channel width (*w*) and suspending medium. Blood suspended in RPMI 1640 medium (*H_o_* = 20%), Dextran 500 (*H_o_* = 20%) or plasma (*H_o_* = 20%) was perfused through the channel array of three different widths (25 µm, 50 µm and 75 µm) and the number of cells (expressed as a percentage of the total passing WBCs) contacting side walls was determined at three locations along the channel length. The measurements were made across 3–5 channels at a flow rate (Q) of 10 µL/hr. A) representative image of WBCs traveling near the walls after margination. B) Near the inlet of the channel (0.05 mm from entrance), 10 to 40% cells contact the wall. As the width increases, the number decreases. There is no significant effect of suspending medium in this region. C) In 25 µm wide channels, margination plateaus near the middle of the channel. D) In 50 µm channels, margination increases nearly linearly with distance from the inlet. E) In 75 µm channels, margination is less efficient, with many WBCs still in the bulk flow at 5 mm from the inlet.

We perfused blood through arrays of straight channels and determined margination at three different distances from the entrance (0.05, 3.10, and 4.95 mm; Figure 2). By using a combination of bright field and fluorescent microscopy, we could image the RBCs, channel walls and WBCs simultaneously (Figure 2A and Video S1). Upon entering the test channels, 20 to 50% of the WBCs already traveled near the wall, and this was not influenced by the suspending medium (Figure 2B). In this entrance region, there was a trend toward more margination as channel width (*w*) increased for the RPMI sample. Analyzing the results farther downstream revealed three general trends for margination (Figure 2C–E):

1. The level of aggregation supported by the suspending media affected the ability of the WBCs to marginate: in general, samples suspended in RPMI 1640 (which had less aggregation; Figure 1C), had less margination, and the most physiologically-representative samples –blood reconstituted in plasma – had higher levels of margination. This suggests that the aggregation characteristics of whole blood are well-tuned to deliver WBCs to post-capillary venule walls. In most cases, Dextran 500 induced levels of margination similar to those in plasma. This result is consistent with reports that blood constituted in aggregating medium induces higher levels of margination in glass tubes, large rectangular channels and real vessels [9]–[11], [13].
2. In general, margination decreased with channel width and increased along the distance away from the inlet (Figures 2C–E). This result is consistent with the work of Goldsmith and Spain [6] who showed that leukocyte margination in glass tubes increased by 20% as they decreased diameter from 155 µm to 100 µm.
3. Some samples –especially those with non-aggregating medium– had very little increase in margination moving along the channel length. This was due to lateral dispersion in these samples, WBCs often changed streamlines, moving toward–as well as away from – the side walls. Thus, a steady state was reached in which lateral WBC dispersion dominated, likely due to wall inertial forces [41] or a lack of development of an organized core of RBCs.

**Figure 3:**
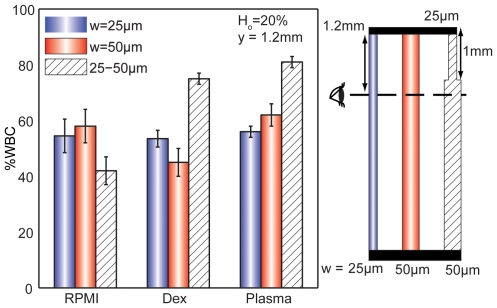
WBC margination varies with blood hematocrit. In plasma, when *y* = 4.95 mm and *H_o_* = 20%, straight channels exhibit maximum WBC margination. Lower (*H_o_* = 10%) and higher (*H_o_* = 40%) results in poor margination

Next, we varied the hematocrit of blood suspended in plasma and measured margination 4.95 mm downstream of the channel inlet (Figure 3). In these studies, there were three major trends:

1. At *H_o_* = 40%, there was a decrease in margination with increasing channel width (*p*<0.01).
2. At *H_o_* = 20%, extensive margination was observed for all channel widths (77.8–95.6%).
3. The trend at low *H_o_* (10%) was similar to that at high *H_o_*, with smaller channels supporting more margination (*p*<0.01). Aggregation was inhibited in these dilute suspensions, and the RBCs failed to organize into a substantial core to exclude the WBCs. In this setting, we expect the leukocyte motion to be dominated by Segre-Silberberg inertial forces [41].

Similar results were observed by Goldsmith and Spain [6]. They found that margination was higher at 20% hematocrit compared to 40%. However, our results contradict with Abbitt *et. al* [13] in large glass channels and *in vivo* observation by Firrell *et. al* [42] who found that margination was invariant when hematocrit was less than 50%.

### Margination at expansions

**Figure 4:**
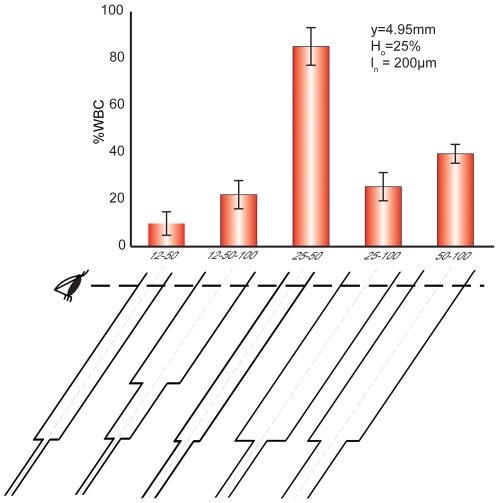
The %WBCs contacting the wall may depend on channel geometry. Comparison of various sudden expansion device geometries show that when *H_o_* = 25%, *l_n_* = 200 µm and *y* = 4.95 mm, blood suspended in plasma has the most dramatic WBC margination for a 25–50 µm sudden expansion.

Previous studies have shown that step changes in conduit size can affect WBC margination [4], [5], [22]. To test this in rectangular microchannels with physiologically comparable sizes and hematocrits, we made devices with step-changes in width from 12 to 50 µm, 12 to 50 to 100 µm, 25 to 50 µm, 25 to 100 µm and 50 to 100 µm (Figure 4). The most effective margination occurred just downstream of 25 to 50 µm expansions. The non-linear behavior in the various expansions is likely due to the reliance of margination on dynamic reorganization and aggregation of the RBCs as the fluid shear rate drops in the expansion. This process is sensitive to the absolute shear rate, as well as the change due to the expansion, RBC aggregation and hematocrit. In our pseudo-2D geometry, the process occurred most efficiently in the 25–50 µm expansion; these widths are larger than measured values for normal capillary-postcapillary diameters (8–15 µm), but are consistent with dimensions measured during inflammation (30–40 µm) [43]. It has been proposed that venule dilation during inflammation has evolved to aid in WBC margination [43], [44]. Our results in rectangular microchannels support this concept.

**Figure 5:**
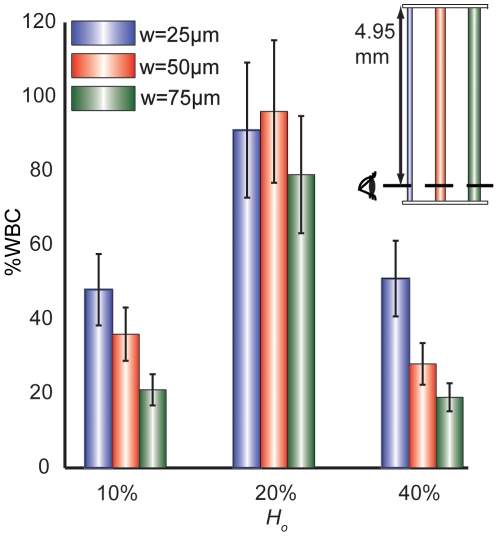
Post-capillary expansions improve WBC margination. In plasma or with dextran 500, when *H_o_* = 20% and *y* = 1.2 mm, WBC margination is improved in a 25–50 µm sudden expansion compared to a 25 µm or 50 µm straight channel. However, in RPMI 1640 where blood aggregation is not significant, straight channels exhibit better margination.

To test the hypothesis that expansion geometries support more margination than straight channels, we directly compared margination in straight (25 µm and 50 µm) and expanding (25–50 µm) channels (Figure 5). These experiments were conducted with blood *H_o_* = 20% and measurements were made 1.2 mm downstream of the channel inlet just below the narrow section (1 mm) of the expansion. In blood suspended in RPMI 1640, straight channels exhibited better margination than the expansion (*p* = 0.03). However, when blood was allowed to aggregate (i.e., in dextran or plasma), there was a significant improvement of margination in the expansion compared to a straight channel (*p*<0.01). This result is only in partial agreement with Schmid-Schönbein and coworkers [5] who showed that sudden expansions with aspect ratios of 1.5 and 1.7 promote more margination than straight tubes. In their experiments, the hematocrit was very low, white cells were simulated as spheres and red cells as disks and their results depended on the orientation of contact between the disks and sphere. Computational studies by Sun *et. al.* [4], [45] and *in vivo* observations by Pearson and coworkers [10], [11] also suggest that post-capillary geometries promote margination.

### Effect of shear rate

**Figure 6:**
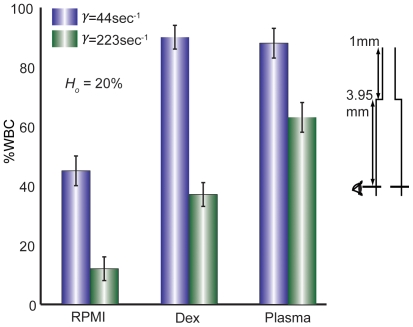
WBC margination is more dramatic at lower shear rates. Blood suspended in RPMI, Dextran 500 or plasma experiences a significant drop in WBC margination as shear rate is increased from 44 sec^−1^ to 223 sec^−1^.

Margination in pseudo-2D sudden expansions is affected by shear rate along the width (*γ_w_*; Figure 6). In our rectangular microchannels, average shear rate along the width of the channel was calculated as 
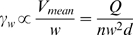
 where *n* is the number of channels in the array (10) and *d* is the depth of the channel (10 µm). The channels with high shear rate exhibited low margination in all three suspension media (*p*<0.01). This result is consistent with previous studies in tubes and vessels [6], [9]–[11] showing lower shear rates support more margination. Again, we observed better margination in aggregating samples (plasma, Dextran), but high shear rates inhibited aggregation and margination. Dispersion of the independent cells in the flow prevented their organization into a central “core” to exclude the WBCs.

### Effect of channel depth in sudden expansions

**Figure 7:**
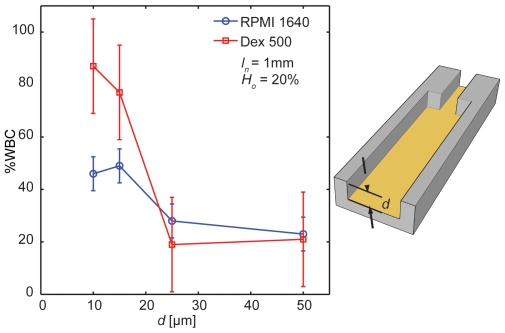
WBC margination varies with channel depth. As the depth of the device is increased above ∼20 µm, margination to the lateral side walls drops off dramatically. This is likely due to the fact that the WBCs are ∼10 µm in diameter, and when the channels exceed 20 µm in height, they are no longer confined to a pseudo-2D geometry. Thus, in higher channels, there is likely margination to all 4 channels walls rather than just the two lateral walls seen in channels with small height.

To assess how the channel aspect ratio affects WBC margination, we fabricated sudden expansions in PDMS devices of varying channel depth, *d* = 10 µm, 15 µm, 25 µm and 50 µm, and quantified margination at the two sidewalls. For devices with walls higher than 15 µm, lateral margination was reduced (Figure 7). This is likely due to margination of WBCs in the *z* direction, toward the top and bottom surfaces; these would not be counted as “marginated” in our analysis, which considered the lateral walls only. Thus, devices with height on the order of a WBC diameter (typically between 8 and 15 µm [46]) restrict vertical dispersion of the WBCs, forcing them laterally instead.

## Discussion

Although some groups have used PDMS devices for blood flow studies, [31]–[33], [47]–[51], there is a lack of quantitative blood rheology data in these new devices. To help define the advantages and limitations of rectangular microchannels in studying blood fluid dynamics, we have quantified WBC margination in PDMS rectangular microchannels under various conditions.

**Figure 8:**
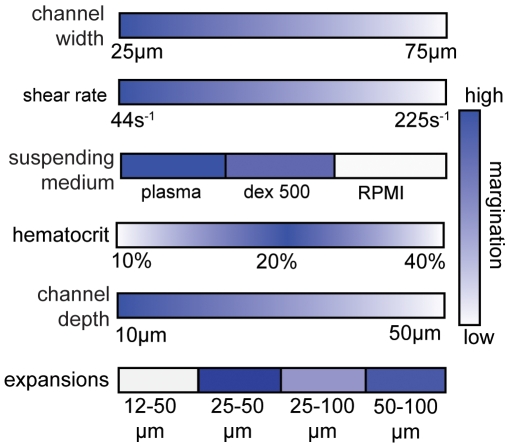
Summary of margination trends in microchannels. Margination depends on channel width (*w*), shear rate (*γ_w_*), RBC aggregation induced by suspending medium, hematocrit (*H_o_*), channel depth (*d*) and sudden expansion geometries. Darker color indicates more margination.

Our experiments show that RBC aggregation and conduit expansions encourage WBC margination and verify that important aspects of blood rheology are retained in microfluidic channels. An illustration of the general trends is shown in Figure 8. Our results are in accordance with many past studies [5], [6], [9]–[11], [14], [42], but absolute values vary–even within the existing literature–due to intrinsic variations in blood properties and experimental conditions. We found that blood suspended in an aggregating medium (plasma or Dextran 500) allows WBCs to marginate most effectively (Figure 2). There is an optimum hematocrit (*H_o_* = 20%) wherein WBC margination is higher than 90% when suspended in plasma or dextran 500 (Figure 3). A low (*H_o_* = 10%) or a high (*H_o_* = 40%) hematocrit results in less margination. Change in hematocrit alters wall shear rate and the RBC velocity profile [52]. A very low or high shear rate at the wall may cause the marginated WBCs to lose the adhesion to the wall. However, in the pseudo 2D geometry, the wall shear rates are different along the width and depth of the channel and the margination phenomenon is more complex. Sudden expansion from 25–50 µm improves WBC margination when blood is suspended in an aggregating medium (Figures 4 and 5). This suggests that postcapillary expansions have evolved to enhance margination. Our results show that high shear results in poor margination (Figure 6). High shear stress may break RBC-RBC bonds and reduce aggregation, thus, inhibiting WBC margination. These aspects of RBC, WBC and plasma flow in microchannels require further investigation. Finally, the artificial pseudo-2D geometry also enhances WBC margination in rectangular microchannels because cell motion is restricted to the x-y plane (Figure 7).

From the perspective of blood rheology and margination in real microvessels, our findings have significant implications. For example, in normal vasculature, network geometry and topology are optimized for delivery of oxygen, nutrients and immune cells. In pathologies such as cancer, the vessels that form have abnormal structure, without defined capillaries and venules. Thus, it is likely that WBC trafficking is impaired in such tissue [4], [53]. Our results also suggest that patients with anemia or who receive anti-coagulants may have altered leukocyte trafficking [54], [55].

Microfabrication technology has advanced rapidly in the past decade; it is now a common tool in many laboratories, used for various applications in science and technology [56]–[60]. Microfluidic devices provide a flexible platform for studying blood flow at the size scale of the microvasculature and for designing analytical devices able to work with many kinds of cell suspensions [58], [61]–[63], [64]. They have some distinct advantages that may be exploited in studies of blood rheology including specifiable, complex network geometry at the correct scale, non-rigid walls that can be functionalized with proteins or cells, high oxygen permeability and low sample volume.

However, PDMS microchannels also have limitations that must be considered for blood flow analyses. For example, when perfusing blood through microfluidic devices, shear rate is an important parameter. Soft lithography-based PDMS devices typically have channels with rectangular cross section, so the network lies within a pseudo-2D geometry. In these low aspect ratio channels, shear rates in the vertical direction (*γ_z_*) can be much higher than those within the plane of the device (*γ_w_*). Since blood cells can be damaged or activated by high shear stresses, the average shear in the vertical (*z*) direction is limiting, and determines the maximum allowable flow rate. At very high flow rates (>2 µl/hr per channel; *γ_z_* ∼600 sec^−1^ [65]), shear forces may damage the cells, leading to fouling and occlusion of channels. On the other hand, low flow rates can also introduce problems. At low flow rates (<0.5 µl/hr per channel; *γ_z_* ∼150 sec^−1^), cell sedimentation and sludging become significant, causing spatial and temporal variations in hematocrit in the experimental system. A number of steps can be taken to minimize these problems. For example, by working with arrays of parallel channels, flow changes in any given segment are not greatly affected if other, parallel paths are occluded, and hematocrit variation can be minimized by decreasing the inlet tubing length and mixing the sample reservoir frequently.

On the other hand, the pseudo-2D geometry of microchannels may provide an advantage for many existing or future applications. For example, the transparency of PDMS and the low channel height result in excellent optical properties for videomicroscopy. And in the current study, we found that WBC margination is enhanced in this geometry, potentially allowing development of a new class of devices that separate and extract WBCs and other rare cells from whole blood without the need for complex 3D fabrication [34].

## Materials and Methods

### Microdevice Design

We designed straight channel and sudden expansion channel arrays. There were 10 parallel channels in each 5 mm long array. All experiments were conducted with channels that were 10 µm deep (*d*) except for those in Figure 7, which included 15 µm, 25 µm and 50 µm deep channels. We used arrays of parallel channels to allow higher pump flow rates and minimize the effect of clogging in a few of the channels.

### Microfabrication

The channel array devices were designed using AutoCAD software (AutoDesk Inc, San Rafael, CA). The Mylar transparencies were printed on a 50,000 dpi resolution printer (Fineline Imaging, Colarado Springs, CO) and were taped to a blank glass plate using Kapton tape. We used SU8-2010, 2025 and 2075 (MicroChem. Corp., Newton, MA) master templates fabricated on Si (100) wafers (University Wafer Corp., Boston, MA) using photolithography. The photolithography system used was a Karl Suss MA 6 mask aligner (Suss MicroTec, Waterbury Center, VT). The devices were fabricated using soft lithography of PDMS [66], [67]. Slygard 184 PDMS prepolymer (Dow Corning, Midland, MI) was cast on the silanized master which had the positive relief of the channel features formed by the SU8 photoresist. The PDMS was then cured at 80°C in a convection oven for 50 minutes. The cured PDMS was peeled off the master and permanently bonded to a 500 µm high PDMS coated glass slide after treating both with oxygen plasma (Harrick Plasma, Ithaca, NY). The microchannel device was flushed with 1% PEG-Silane (O-methyl-O'[2-(trimethoxysilyl)ethyl)]polyethylene glycol, MW 5000; Shearwater Polymers Inc., Huntsville, AL) for 20 min, followed by perfusion with PBS buffer for 5 min prior to use.

### Blood Preparation

Blood was obtained from buffy coats collected at the Massachusetts General Hospital blood bank and used within 48 hours. Any changes in blood rheology caused by blood storage conditions did not significantly affect WBC margination: freshly drawn blood and blood stored at 4°C for 48 hours supported the same levels of margination in 50 and 75 µm channels (Figure S1). The hematocrit was measured using an Autocrit Ultra3 hematology analyzer (Becton Dickinson, Franklin Lakes, NJ). WBCs were stained using the fluorescent dye Carboxyfluorescein succinimidyl ester (CFSE; Invitrogen, Carlsbad, CA) as per the product instructions. Blood was washed three times at 1500 rpm and re-suspended in the required medium (RPMI 1640 buffer, 2% Dextran-500 or plasma) prior to CFSE staining. We removed the platelets from the blood sample using OptiPrep (Fisher Scientific, Pittsburgh, PA) as per product instructions. Mono-nuclear leukocytes were isolated using Ficoll-Paque Plus (GE Life Sciences, Piscataway, NJ) as per instructions and then re-mixed with the RBCs at the required concentration (7000–10000 cells/µl). To prepare low hematocrit samples, erythrocytes were diluted to the desired concentration during the final washing step of the sample preparation.

### Experimental Setup

Figure 1A illustrates the experimental setup. Images were acquired using the 20×objective of an epifluorescence microscope (Nikon Diaphot) and a CMOS Camera (PCO1200s, Cooke Corp., Germany). A syringe pump (Nanomite, Harvard Apparatus, Holliston, MA) with 500 µL syringe (Hamilton Company, Reno, NV) and 23 gauge needle (0.5″ long, type 304, ID 0.017″, OD 0.025″) was used to push the samples through the microfluidic devices. Fluid entered and exited the device through Tygon tubing (ID 0.02″, OD 0.06″, 0.02″ wall; VWR, Brisbane, CA) connected to stainless steel tubes (0.025″ OD x 0.017″ ID, 0.500″ length; New England Small Tube, Litchfield, NH) inserted into the PDMS ports. To minimize the effects of blood sedimentation, we paused the experiment every five minutes to mix the blood sample. In this study, we adjust the flow rate (*Q*) in the array of channels such that *γ_z_*∼400 sec^−1^ except in Figure 6 where we show that margination is affected by shear rate. The flow rate variation in individual channels within the parallel array varied by as much as 15% (Figure S2). However, any variation in margination caused by this small variation in velocity was insignificant compared to that caused by other experimental variables. Occasionally, clogging of channels caused variation in flow rate during the experiment. If more than one channel within the array showed signs of clogging, the experiment was discontinued.

### Statistical Analysis

We computed *p* values of the Student's *t-*test for a level of significance (α) of 0.05 for statistical comparison. The error bars on the figures denote sample standard deviations.

## Supporting Information

Figure S1 Comparison of WBC margination in blood bank blood and freshly drawn blood.
(0.04 MB PDF)

Figure S2 Variation of Flow Rate (*q*) within Microfluidic Channel Array
(0.05 MB PDF)

Video S1 Movie showing Fluorescent WBCs marginated to the sidewalls of a 50 µm wide microchannel (20×magnification). The RBC hematocrit was 20%.
(2.33 MB MOV)

## References

[1] Williamson JR, Grisham JW (1961). Electron microscopy of leukocytic margination and emigration in acute inflammation in dog pancreas.. Am J Pathol.

[2] Munn LL, Melder RJ, Jain RK (1996). Role of erythrocytes in leukocyte-endothelial interactions: mathematical model and experimental validation.. Biophys J.

[3] Alon R, Hammer DA, Springer TA (1995). Lifetime of the P-selectin-carbohydrate bond and its response to tensile froce in hydrodynamic flow.. Nature.

[4] Sun CH, Migliorini C, Munn LL (2003). Red blood cells initiate leukocyte rolling in postcapillary expansions: A lattice Boltzmann analysis.. Biophysical Journal.

[5] Schmid-Schonbein GW, Usami S, Skalak R, Chien S (1980). The interaction of leukocytes and erythrocytes in capillary and postcapillary vessels.. Microvasc Res.

[6] Goldsmith HL, Spain S (1984). Margination of leukocytes in blood flow through small tubes.. Microvasc Res.

[7] Goldsmith HL, Spain S (1984). Radial distribution of white cells in tube flow.. Kroc Found Ser.

[8] Colditz IG (1985). Margination and emigration of leucocytes.. Surv Synth Pathol Res.

[9] Nobis U, Pries AR, Cokelet GR, Gaehtgens P (1985). Radial distribution of white cells during blood flow in small tubes.. Microvasc Res.

[10] Pearson MJ, Lipowsky HH (2000). Influence of erythrocyte aggregation on leukocyte margination in postcapillary venules of rat mesentery.. Am J Physiol Heart Circ Physiol.

[11] Pearson MJ, Lipowsky HH (2004). Effect of fibrinogen on leukocyte margination and adhesion in postcapillary venules.. Microcirculation.

[12] Nash GB, Watts T, Thornton C, Barigou M (2008). Red cell aggregation as a factor influencing margination and adhesion of leukocytes and platelets.. Clin Hemorheol Microcirc.

[13] Abbitt KB, Nash GB (2003). Rheological properties of the blood influencing selectin-mediated adhesion of flowing leukocytes.. Am J Physiol Heart Circ Physiol.

[14] Bagge U, Blixt A, Strid KG (1983). The initiation of post-capillary margination of leukocytes: studies in vitro on the influence of erythrocyte concentration and flow velocity.. Int J Microcirc Clin Exp.

[15] Bagge U, Karlsson R (1980). Maintenance of white blood cell margination at the passage through small venular junctions.. Microvasc Res.

[16] Phibbs RH (1966). Distribution of leukocytes in blood flowing through arteries.. Am J Physiol.

[17] Bevilacqua MP (1993). Endothelial-leukocyte adhesion molecules.. Annual Review of Immunology.

[18] Tedder TF, Steeber DA, Chen A, Engel P (1995). The selectins - vascular adhesion molecules.. Faseb Journal.

[19] Wang HB, Wang JT, Zhang L, Geng ZH, Xu WL (2007). P-selectin primes leukocyte integrin activation during inflammation.. Nature Immunology.

[20] Iigo Y, Suematsu M, Higashida T, Oheda J, Matsumoto K (1997). Constitutive expression of ICAM-1 in rat microvascular systems analyzed by laser confocal microscopy.. Am J Physiol.

[21] Ley K (1996). Molecular mechanisms of leukocyte recruitment in the inflammatory process.. Cardiovascular Research.

[22] Sun CH, Munn LL (2006). Influence of erythrocyte aggregation on leukocyte margination in postcapillary expansions: A lattice Boltzmann analysis; 2006.

[23] Brenner H (1961). The slow motion of a sphere through a viscous fluid towards a plane surface.. Chemical Engineering Science.

[24] Schmid-Schonbein H, Gaehtgens P, Hirsch H (1968). On the shear rate dependence of red cell aggregation in vitro.. J Clin Invest.

[25] Barbee JH, Cokelet GR (1971). Prediction of blood flow in tubes with diameters as small as 29 microns.. Microvasc Res.

[26] Barbee JH, Cokelet GR (1971). The Fahraeus effect.. Microvasc Res.

[27] Goldsmith HL (1971). Red cell motions and wall interactions in tube flow.. Fed Proc.

[28] Goldsmith HL (1971). Deformation of human red cells in tube flow.. Biorheology.

[29] Lawrence MB, McIntire LV, Eskin SG (1987). Effect of flow on polymorphonuclear leukocyte endothelial-cell adhesion.. Blood.

[30] Munn LL, Melder RJ, Jain RK (1994). Analysis of cell flux in the parallel plate flow chamber: implications for cell capture studies.. Biophys J.

[31] Lima R, Wada S, Tanaka S, Takeda M, Ishikawa T (2008). In vitro blood flow in a rectangular PDMS microchannel: experimental observations using a confocal micro-PIV system.. Biomed Microdevices.

[32] Faivre M, Abkarian M, Bickraj K, Stone HA (2006). Geometrical focusing of cells in a microfluidic device: an approach to separate blood plasma.. Biorheology.

[33] Shevkoplyas SS, Gifford SC, Yoshida T, Bitensky MW (2003). Prototype of an in vitro model of the microcirculation.. Microvasc Res.

[34] Shevkoplyas SS, Yoshida T, Munn LL, Bitensky MW (2005). Biomimetic autoseparation of leukocytes from whole blood in a microfluidic device.. Anal Chem.

[35] Davis JA, Inglis DW, Morton KJ, Lawrence DA, Huang LR (2006). Deterministic hydrodynamics: Taking blood apart.. Proceedings of the National Academy of Sciences of the United States of America.

[36] Jaggi RD, Sandoz R, Effenhauser CS (2007). Microfluidic depletion of red blood cells from whole blood in high-aspect-ratio microchannels.. Microfluidics and Nanofluidics.

[37] VanDelinder V, Groisman A (2007). Perfusion in microfluidic cross-flow: Separation of white blood cells from whole blood and exchange of medium in a continuous flow.. Analytical Chemistry.

[38] Kim S, Popel AS, Intaglietta M, Johnson PC (2005). Aggregate formation of erythrocytes in postcapillary venules.. Am J Physiol Heart Circ Physiol.

[39] Kim S, Zhen J, Popel AS, Intaglietta M, Johnson PC (2007). Contributions of collision rate and collision efficiency to erythrocyte aggregation in postcapillary venules at low flow rates.. Am J Physiol Heart Circ Physiol.

[40] Neu B, Wenby R, Meiselman HJ (2008). Effects of dextran molecular weight on red blood cell aggregation.. Biophys J.

[41] Segre G, Silberberg A (1962). Behaviour of macroscopic rigid spheres in Poiseuille flow Part 2. Experimental results and interpretation.. Journal of Fluid Mechanics.

[42] Firrell JC, Lipowsky HH (1989). Leukocyte margination and deformation in mesenteric venules of rat.. Am J Physiol.

[43] Secomb TW, Konerding MA, West CA, Su M, Young AJ (2003). Microangiectasias: Structural regulators of lymphocyte transmigration.. Proceedings of the National Academy of Sciences of the United States of America.

[44] Schmid-Schonbein GW (2006). Analysis of inflammation.. Annual Review of Biomedical Engineering.

[45] Sun C, Munn LL (2005). Particulate nature of blood determines macroscopic rheology: a 2-D lattice Boltzmann analysis.. Biophys J.

[46] Skalak R, Chien S (1987). Handbook of bioengineering..

[47] Shevkoplyas SS, Yoshida T, Gifford SC, Bitensky MW (2006). Direct measurement of the impact of impaired erythrocyte deformability on microvascular network perfusion in a microfluidic device.. Lab on a Chip.

[48] Yang XH, Long L, Wang KM, Liu HM, Li ML Direct measurement of impaired erythrocyte deformability on an inter-channel microfluidic device..

[49] Higgins JM, Eddington DT, Bhatia SN, Mahadevan L (2007). Sickle cell vasoocclusion and rescue in a microfluidic device.. Proceedings of the National Academy of Sciences of the United States of America.

[50] Rosenbluth MJ, Lam WA, Fletcher DA (2008). Analyzing cell mechanics in hematologic diseases with microfluidic biophysical flow cytometry.. Lab on a Chip.

[51] Wan JD, Ristenpart WD, Stone HA (2008). Dynamics of shear-induced ATP release from red blood cells.. Proceedings of the National Academy of Sciences of the United States of America.

[52] Tangelder GJ, Slaaf DW, Arts T, Reneman RS (1988). Wall shear rate in arterioles in vivo: least estimates from platelet velocity profiles.. Am J Physiol.

[53] Less JR, Skalak TC, Sevick EM, Jain RK (1991). Microvascular architecture in a mammary carcinoma: branching patterns and vessel dimensions.. Cancer Res.

[54] Deitcher SR Cancer and thrombosis: Mechanisms and treatment; 2003 May 08-10;.

[55] Vlodavsky I, Ilan N, Nadir Y, Brenner B, Katz BZ Heparanase, heparin and the coagulation system in cancer progression; 2007 Oct 26–28; Bergamo, ITALY..

[56] Whitesides GM, Ostuni E, Takayama S, Jiang XY, Ingber DE (2001). Soft lithography in biology and biochemistry.. Annual Review of Biomedical Engineering.

[57] Dittrich PS, Tachikawa K, Manz A (2006). Micro total analysis systems. Latest advancements and trends.. Analytical Chemistry.

[58] Weibel DB, Whitesides GM (2006). Applications of microfluidics in chemical biology.. Current Opinion in Chemical Biology.

[59] Weigl B, Domingo G, LaBarre P, Gerlach J (2008). Towards non- and minimally instrumented, microfluidics-based diagnostic devices.. Lab on a Chip.

[60] West J, Becker M, Tombrink S, Manz A (2008). Micro total analysis systems: Latest achievements.. Analytical Chemistry.

[61] Chen X, Cui DF (2009). Microfluidic devices for sample pretreatment and applications.. Microsystem Technologies-Micro-and Nanosystems-Information Storage and Processing Systems.

[62] Chen P, Feng XJ, Du W, Liu BF (2008). Microfluidic chips for cell sorting.. Frontiers in Bioscience.

[63] Toner M, Irimia D (2005). Blood-on-a-chip.. Annual Review of Biomedical Engineering.

[64] Andersson H, van den Berg A (2003). Microfluidic devices for cellomics: a review.. Sensors and Actuators B-Chemical.

[65] Shah RK, London AL (1978). Laminar Flow Forced Convection in Ducts..

[66] Duffy DC, McDonald JC, Schueller OJA, Whitesides GM (1998). Rapid prototyping of microfluidic systems in poly(dimethylsiloxane).. Analytical Chemistry.

[67] Jain A, Munn LL, Herold KE, Rasooly A (2009). Design and Fabrication of Microfluidic Devices for Flow-based Separation of Blood Cells.. Lab-on-a-Chip Technology.

