# Supplementary material for: Determinants of Leukocyte Margination in Rectangular Microchannels
Source: PLoS One. 2009 Sep 21;4(9):e7104. doi: 10.1371/journal.pone.0007104 (PMC2740820; doi:10.1371/journal.pone.0007104)
Supplement: Figure S1 — Comparison of WBC margination in blood bank blood and freshly drawn blood. (0.04 MB PDF) [file pone.0007104.s001.pdf]

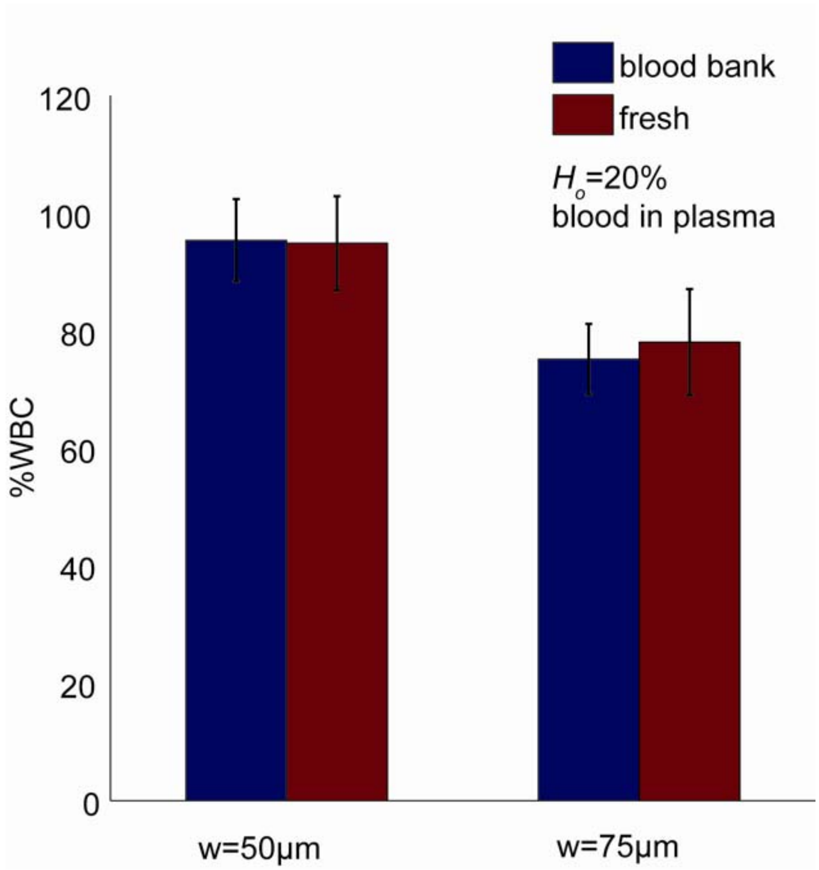

Figure S1. WBC margination in freshly drawn and 48 hour-old banked blood stored at 4°C in 50 and 75 μm channels.
