# Supplementary material for: Determinants of Leukocyte Margination in Rectangular Microchannels
Source: PLoS One. 2009 Sep 21;4(9):e7104. doi: 10.1371/journal.pone.0007104 (PMC2740820; doi:10.1371/journal.pone.0007104)
Supplement: Figure S2 — Variation of Flow Rate (q) within Microfluidic Channel Array (0.05 MB PDF) [file pone.0007104.s002.pdf]

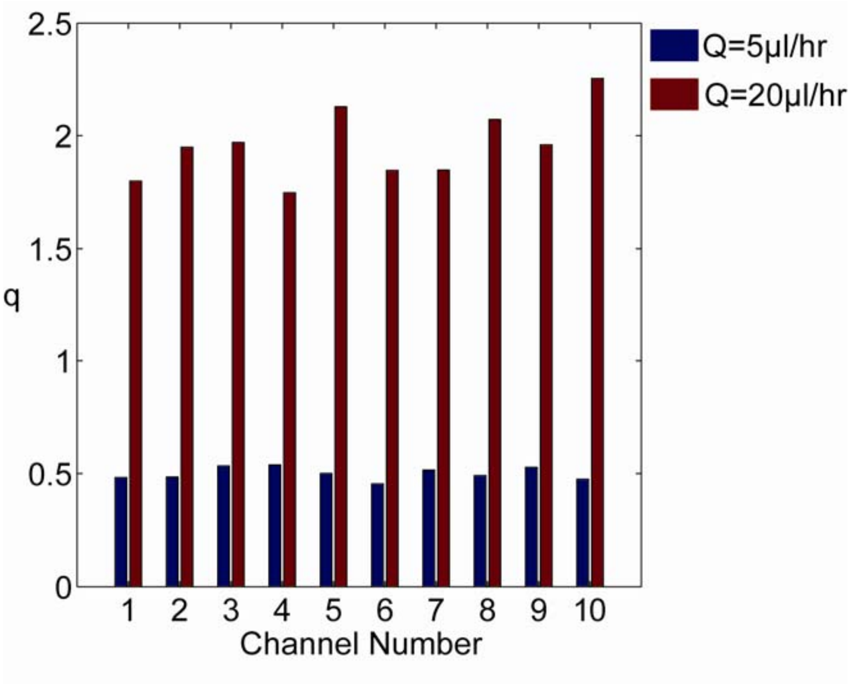

Figure S2. Channel flow rates (*q*) for 2 different input flow rates (*Q*) in an array of ten 50μm wide channels.
